# Face mask sampling for the detection of microbes in expelled aerosols and the impact of airway clearance on microbial yield in children with cystic fibrosis: a feasibility trial

**DOI:** 10.1099/jmm.0.002150

**Published:** 2026-04-08

**Authors:** Naomi A. Dayman, Deepa Patel, Anika Wisniewska, Eve Fletcher, Molla Imaduddin Ahmed, Deborah Modha, Michael R. Barer, Erol A. Gaillard

**Affiliations:** 1 Department of Respiratory Sciences, Institute for Lung Health, Leicester NIHR Biomedical Research Centre, University of Leicester, Leicester LE1 7RH, UK; 2 Leicester Royal Infirmary, University Hospitals of Leicester, Infirmary Square, Leicester LE1 5WW, UK; 3 University of Leicester, University Road, Leicester LE1 7RH, UK

**Keywords:** airway clearance, cystic fibrosis, face mask sampling

## Abstract

**Background.** Early identification of pulmonary exacerbations is vital for the management of cystic fibrosis (CF). Non-invasive airway sampling in preschool children can be inaccurate. Face mask sampling (FMS) is a novel non-invasive approach that can be used to assess microbial airway pathogens in patients with CF.

**Methods.** Prospective cross-sectional study in children with CF. Children wore a suitably sized face mask fitted with two strips of a polyvinyl alcohol sampling matrix for a period of 15 min. Routine microbiology sampling using cough swab, sputum and/or bronchoalveolar lavage was completed following FMS. Children then completed their routine airway clearance with their physiotherapist. Following this, a separate face mask was worn for a further 15 min, after which further routine microbiology sampling (cough swab or sputum) was completed. The face masks were stored at room temperature before transfer and processing in the laboratory to quantify bacterial burden and identify key pathogens such as *Mycobacterium abscessus* and *Pseudomonas aeruginosa*.

**Results.** Eleven children (six male, median age 12 years, range 1–16 years), from the Leicester CF cohort were included. All patients tolerated the FMS. Nine face mask samples from 11 participants isolated respiratory pathogens, including *M. abscessus* (*n*=3). *P. aeruginosa* was not detected on face mask samples. There was a trend towards an increase in microbial yield (prGen16s) following airway clearance (*n*=5), but this did not reach statistical significance.

**Conclusions.** FMS systems are feasible for children and young people with CF. They may provide an effective method to detect exhaled lower airway pathogens including non-tuberculous mycobacteria. The effect of physiotherapy on the exhaled microbiome needs to be explored further.

**Impact Statement**

Young children with cystic fibrosis (CF) are often asymptomatic, cough-free and non-sputum producers. This poses a significant challenge to the detection of lower airway microbial infection and/or colonization with problematic respiratory pathogens. Early identification of pulmonary exacerbations is vital for the management of CF. Non-invasive airway sampling in preschool children can be inaccurate, and methods such as cough swabs are ineffective at detecting organisms such as non-tuberculous mycobacteria (NTM). Face mask sampling (FMS) is a novel non-invasive approach using strips of a sampling matrix presented within a mask to capture exhaled microbes including NTM. FMS in children and young people and non-productive patients with CF could be an alternative way to non-invasively detect microbes from the lower airway.

## Introduction

Early identification of respiratory pathogens is critical to deliver timely targeted treatment in participants with cystic fibrosis (CF) [1]. Early detection of problem organisms such as *Pseudomonas aeruginosa* and non-tuberculous mycobacteria (NTM) is essential to reduce the risk of spread in the clinic setting and to increase the chances of successful eradication [2,4].

Children and young people (CYP) with CF, especially those on modulator treatment, are often non-sputum producers [5], and widely used non-invasive sampling such as cough swabs is ineffective [6] at detecting organisms such as NTM [1].

Face mask sampling (FMS) is a novel non-invasive approach to sample exhaled lower airway microbes [7,8]. Williams *et al*. have demonstrated that exhaled *Mycobacterium tuberculosis* from both suspected and diagnosed cases of tuberculosis is linked with household transmission of infection [9,11].

Our study aims were to investigate (1) the feasibility of non-invasive FMS in CYP (feasibility was defined by the ability of CYP with CF to tolerate the mask for a minimum period of time, set at 15 min based on previous research and the ability to [12] extract material for laboratory analysis) and (2) whether physiotherapy treatment altered the exhaled microbiome with greater detection of specific organisms such as *P. aeruginosa* and NTM.

## Theory and implementation

### Participants

Prospective cohort feasibility study in CYP with a confirmed diagnosis of CF (two CF-causing mutations and/or a sweat chloride level of >60 mmol l^−1^). We recruited CYP aged 0–18 years at the Leicester Children’s Hospital between November 2017 and March 2018 during routine clinical reviews. Participants were prioritized if they had a bronchoscopy or were sputum producing to be able to pair with FMS. This was done opportunistically due to limited numbers of available masks. Participants needed to tolerate face mask wearing for 15 min. Informed written consent was obtained from participants and/or carers. The study was approved as part of the Leicester longitudinal study of respiratory infections and microbiomics in CF by the East Midlands Research Ethics Committee, reference number 12/WM/0285.

### Face mask sampling

Routine airway sampling was completed first (cough swab or sputum) and submitted for routine microbiological analysis. Subsequent FMS followed the principles previously described [10,11]. Briefly, a standard appropriately sized polyvinyl chloride oxygen delivery face mask with two inserted strips of a polyvinyl alcohol (PVA) sampling matrix (~5 cm × 2 cm) was applied. The modified face mask was worn with a respiratory specialist physiotherapist present for 15 min in a hospital clinic room. The 15 min was chosen because previous face mask data suggests that 15 min is a good compromise between microbial yield and patient comfort [12]. CYP were instructed to maintain normal tidal breathing but were free to sit down or walk around the room. Following the 15-min period, the ‘pre-physiotherapy’ face mask was removed and placed into a labelled transport bag. The CYP subsequently completed their routine airway clearance supported by their physiotherapist to mobilize secretions. A second routine airway sample was obtained at the end of airway clearance for microbiological analysis. FMS was then repeated with a different face mask for another 15 min, providing a ‘post-physiotherapy’ sample. Routine airway samples were taken to the laboratory for processing within 24 h.

After sampling, face masks were placed, still open, in double plastic bags. The bags were left with a small opening until the exposed strips were observed to be dry, the mask was then flattened and the bags were double sealed and batch stored at room temperature.

FMSs were processed within 1 month of sampling. Stability of PCR signals from stored *Mycobacterium*-contaminated PVA strips is shown in Table 1 and confirms that loss of positivity was unlikely to have affected our results. From exposed masks, the two PVA strips were dissolved in 4.5 ml Tris buffer (25 mM, pH 8.0) and then centrifuged at 20,000 ***g*** for 10 min, and the pellet was resuspended in Tris-EDTA buffer (comprising the pH buffer Tris and the cation chelator EDTA). The suspension was stored at −80°C. Cells were disrupted by bead-beating and DNA extraction based on the methods described by Reddy and colleagues [10,11, 13]. Extracts were analysed using PCR. For genus-level quantitation of mycobacteria and for *Mycobacterium abscessus* detection/quantitation, the methods, primers and conditions described by Chae and colleagues [14] were used. This involved targeting the mycobacterial 16S rDNA gene and *M. abscessus* (mass_3219). Sanger sequencing was used to confirm the identity of the mycobacterial 16S amplicons in which a positive *M. abscessus* signal was obtained. In addition, universal bacterial 16S rDNA, *P. aeruginosa* and *S. aureus* and assays were also run to assess bacterial yields in the samples [15].

**Table 1.** Stability of PCR signals from stored PVA strips

Day	Mean Ct±sd
0	21.00±0.34
7	21.40±0.17
30	21.83±0.40*
60	21.97±0.35*

PVA strips were contaminated with a fixed inoculum of *M.tuberculosis* H37Rv (attenuated strain kindly provided by Dr WR Jacobs [20]) then stored dry at room temperature for the periods shown. DNA extracts were prepared as described in methods and these were assayed by IS6110-direct PCR in triplicate as previoulsy described [10] Minor but significant (*-p<0.05) signal decline is shown at 30 and 60 days.

## Data Summary

### Data analysis

Wilcoxon signed-rank test was used to analyse paired quantitative data in this study. No statistical difference was detected in bacterial yield or mycobacterial yield with the novel face mask sampling (FMS) and completing airway clearance techniques (ACTs).

### Results

#### Cohort characteristics

Eleven children [six male; six homozygous F508, three heterozygous; mean age 12 years (range 1–16 years); mean FEV_1_ (*n*=10) 77% (range 58–92%); median body mass index 17.3 kg m^−2^ (range 13.34–29.5 kg m^−2^)] participated in this study.

#### Microbiological status

- Non-tuberculous mycobacteria (NTM): Eight were NTM naïve (73%), two had previously isolated NTM (*Mycobacterium abscessus*/*Mycobacterium intracellulare*) (>2 years previously, 18%) and one had re-isolated *M. abscessus* in the previous month.
- *Pseudomonas aeruginosa*: Two were naïve, eight had transient isolations and one was chronically colonized.
- Other respiratory pathogens: Seven participants had previously isolated *Staphylococcus aureus*, including two in the year prior to the study.

#### Microbiological findings

A summary of all respiratory microbiological samples pre-/post-physiotherapy is shown in Table 2. Three non-sputum-producing participants managed to produce a sputum sample after physiotherapy.

**Table 2.** Summary of upper and lower airway samples and face mask samples taken and routine airway clearance techniques used by the patient with CF in this study

Patient ID	Physiotherapy adjunct	Pre-physiotherapy: sputum (Sp) or cough swab (Cs) sample		Post-physiotherapy: sputum (Sp) or cough swab (Cs) sample	
		Sputum/cough swab	Face mask sampling	Sputum/cough swab	Face mask sampling
CF1	Clearway	Cs – NRF	*Mycobacterium* 16S detected	Cs – NRF	*Mycobacterium* 16S detected
CF2	Pari PEP	Cs – NRF	*Mycobacterium* 16S detected	Cs – NRF	*Mycobacterium* 16S detected
CF3	Pari PEP/autogenic drainage	Sp – *Achromobacter* species, *Candida*, *M. gordonae*	*Mycobacterium* 16S detected	Sp – *Candida*, *Achromobacter* species, NRF	*Mycobacterium* 16S detected
CF4	Pari PEP/autogenic drainage	Sp – NRF	*Mycobacterium* 16S detected *S. aureus*	Sp – *Aspergillus fumigatus*/*Candida*	*Mycobacterium* 16S detected *S. aureus*
CF5	Aerobika	Cs – no growth	*Mycobacterium* 16S detected *S. aureus*	Cs – no growth	*Mycobacterium* 16S detected *S. aureus*
CF6	Autogenic drainage	Cs – NRF	***M. abscessus***	Sp – *Aspergillus fumigatus*/*Candida*, NRF	***M. abscessus***
CF7	Nippy clearway in IPPB mode	Cs – no growth	***M. abscessus***	Sp – *Aspergillus fumigatus*/*Candida*, NRF	*Mycobacterium* 16S detected
CF8	Astra PEP	Cs – NRF	*Mycobacterium* 16S detected	Cs – NRF	*Mycobacterium* 16S detected
CF9	Pari PEP	Cs – NRF	***M. abscessus***	Cs – NRF	***M. abscessus***
CF10	Autogenic drainage	Sp – *Ochrobactrum anthropi*, *Aspergillus fumigatus*, *Candida* species	*Mycobacterium* 16S detected	Sp – NTM -ve	*Mycobacterium* 16S detected
CF11	Pari PEP	Cs – NRF	*Mycobacterium* 16S detected	Sp – NRF, *S. aureus*, ***P. aeruginosa***	*Mycobacterium* 16S detected

IPPB, intermittent positive pressure breathing; NIPPY, non-invasive positive pressure ventilation; NRF, normal respiratory flora; NTM, non-tuberculosis mycobacterium; PEP, positive expiratory pressure.

- **Face mask sampling**

**Fig. 1.**
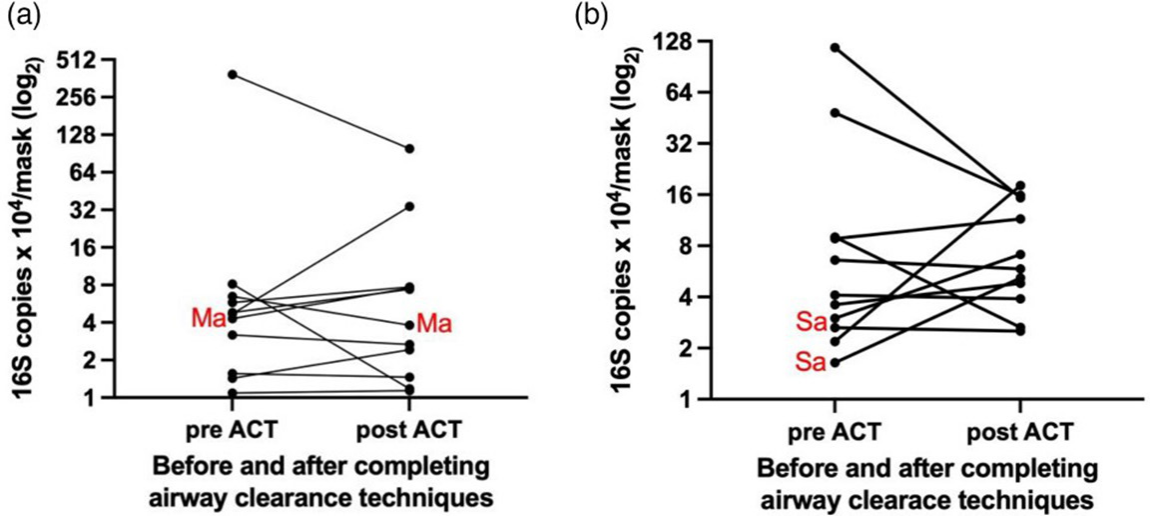
(a) Mycobacterial burden after wearing face mask sample for 15 min before and after airway clearance techniques. (b) Bacterial burden after wearing face mask sample for 15 min before and after airway clearance techniques.

Bacteria including *Mycobacterium* 16S rRNA were detected and quantifiable in the mask samples (Fig. 1a, b). Respiratory pathogens were isolated from nine FMS samples collected from 11 participants. Two children had isolated *M. abscessus* on both pre- and post-face mask samples (Table 2). Both children had previously isolated *M. abscessus,* completed a 1-year eradication regime and were *M. abscessus* negative on standard airway sampling. The other participant had *M. abscessus* detected on the ‘pre-physiotherapy’ mask, but ‘post-physiotherapy’ FMS and sputum were negative. *P. aeruginosa* was not detected. *S. aureus* was detected on the FMS of two participants.

- **Microbial yield**

Five participants had an increase in FMS bacterial yield post-physiotherapy using the 16S rRNA detection methods (Fig. 1b). This was similar for the detection of the *Mycobacterium*-specific 16S rRNA copies (Fig. 1a). There was no general trend relating to microbial yield on FMS before and after ACT. For mycobacterial assays, there was only one similar increase and two declines. By the general 16S assay, three individuals showed >2-fold increases in output and three >2-fold declines.

- **Sputum sampling**

Results are shown in Table 2.

- **Cough swab sampling**

No respiratory pathogens were isolated from cough swabs.

## Discussion

Early detection of problematic bacterial pathogens increases the chances of successful eradication, prevents further lung damage and reduces the risk of epidemic spreading and cross-infection of these pathogens in the clinical setting [16,18]. Current routine airway sampling is insensitive in non-productive patients. Discovering other ways to detect problematic bacteria in CYP in an era of non-productive patients with CF is needed.

To our knowledge, this is the first study exploring the use of FMS in CYP with CF [11]. FMSs were obtained from paediatric patients for 15 min each time. The tolerability of the face masks was assessed by the children keeping it on their face for the 15 min, not causing distress and the older children reporting that the mask did not feel uncomfortable. During this study, the face masks were well tolerated from ages 1–16 years. The long-term goal is to develop routine repeat sampling as a clinical tool. However, the greater the sampling time, the less likely it is that this will be an acceptable tool for routine clinical sampling in paediatrics, and previous work suggests that 15 min is a good compromise between microbial yield and patient comfort. We will need to further validate the comfort of the masks in a larger trial.

Our primary focus was to detect problematic respiratory pathogens. We did detect *M. abscessus* on the FMS but did not detect *P. aeruginosa* using the FMS. There was no consistent direction in the change of the NTM or the bacterial yield pre- and post-physiotherapy on the FMS. In future larger studies, we would like to explore ways in which we could increase the yield, utilizing physiotherapy techniques that have been explored in this study.

Our study limitations include a small sample size. Larger studies are required to investigate the exhaled microbiome in CF using our non-invasive novel FMS technique.

Secondly, we were unable to test if the bacteria were dead or alive. The detection of NTM in the face mask may indicate that these participants are ‘spreaders’, and we hypothesize that, similar to what was shown in FMS studies in tuberculosis [9], participants with NTM in the lower airways but not in the exhaled microbiome may spread the organism less easily. We did not test for fungi in FMS, but this is feasible and should be considered in future studies in CYP with CF [19].

A further limitation was that due to the wide age range, all children completed their individualized routine ACTs. Differing techniques are necessary due to the child’s developmental understanding of their treatment, clinical status and how ACTs can be implemented either by the caregiver or by the child independently. A senior physiotherapist, however, was present for each of the treatments to ensure consistency and effectiveness in their ACT. This was a small feasibility study in patients with CF, and the ACT that the patient was familiar with was continued for this study. A future larger study should address the question of microbial yield stratified by the ACT used by patients.

## Conclusion

FMS systems are feasible for CYP with CF. FMS may provide an effective method to detect problematic exhaled lower airway pathogens including NTM. The effect of physiotherapy on the exhaled microbiome yield also needs to be explored further.

## Abbreviations

ACT: airway clearance technique
CF: cystic fibrosis
CYP: children and young people
FMS: face mask sampling
IPPB: intermittent positive pressure breathing
NIPPY: non-invasive positive pressure ventilation
NRF: normal respiratory flora
NTM: non-tuberculous mycobacteria
PEP: positive expiratory pressure
PVA: polyvinyl alcohol
